# Dual contraception method utilization and associated factors among women on anti-retroviral therapy in public facilities of Bishoftu town, Oromia, Ethiopia

**DOI:** 10.1371/journal.pone.0280447

**Published:** 2023-01-17

**Authors:** Daniel Yohannes Bedecha, Mosisa Assefa Gurmu, Negeso Gebeyehu Gejo

**Affiliations:** 1 Department of Midwifery, School of Health Sciences, Madda Walabu University, Shashemene, Ethiopia; 2 Bishoftu Town Administration Health Office, Oromia Region Health Bureau, Bishoftu, Ethiopia; Medical Research Foundation of Trinidad and Tobago, TRINIDAD AND TOBAGO

## Abstract

**Introduction:**

Dual contraception is a method used to prevent sexually transmitted infections (STIs) including Human Immunodeficiency Virus (HIV) and unintended pregnancies. Prevention of unintended pregnancy in people living with HIV (PLHIV) and ART given to pregnant women to suppress viral load prevent transmission of HIV to children. Counseling and provision of dual contraceptive methods is a very cost-effective method to tackle this double burden among women living with HIV. However, little has been known about utilization of dual methods among HIV positive women in Bishoftu town and its surroundings.

**Objective:**

The aim of this study was to assess dual contraception method utilization and associated factors among reproductive age women who were on antiretroviral therapy in public health facilities of Bishoftu town.

**Methods:**

A facility based cross- sectional study was conducted from November 01 to December 30, 2020. The study participants were selected by using simple random sampling technique. Data were collected using a pretested and structured questionnaire through a face to face interview. Data were processed and analyzed using SPSS version 20. Frequencies and percentages were used to describe characteristics of participants. Bivariable and multivariable logistic regression analyses were used to identify variables which had an independent association with the dependent variable. The degree of association between dependent and independent variables were measured using odds ratio with 95% confidence interval. Level of significance was set at a p-value less than 0.05.

**Results:**

The Magnitude of dual contraceptive utilization of women living with HIV in Bishoftu town was 56.9% (95% Cl (51.6, 62.1). Being married (AOR = 4.33; 95% Cl (1.67, 11.27), not getting pregnant since the start of chronic care follow up (AOR = 2.19; 95% Cl (2.90, 3.70), having a partner positive for HIV (AOR = 2.67; 95% Cl (1.34, 5.32) and having a partner negative for HIV (AOR = 2.38; 95% Cl (1.09, 5.20) were factors independently associated with dual method contraceptive use.

**Conclusion:**

The study showed that use of dual contraceptive methods was low; factors like marital status, partner HIV status, and pregnancy after chronic HIV care follow up were found to be significantly associated with dual contraceptive method use. In addition to ART, use of dual contraceptive methods utilization may play a role in prevention of HIV infection in children and is important in the prevention of unintended pregnancy.

## Introduction

Dual contraceptive utilization refers to the use of a barrier contraceptive (i.e., condoms), which can reduce transmission of many sexually transmitted infections (STIs), and another effective family planning method that can prevent pregnancy (like Depo Provera, Jedelle etc…) [1].

In 2021, 1.5 million [1.1–2.0 million] people contracted HIV, and 650 000 [510 000–860 000] people died from HIV-related causes. At the end of 2021, there were an estimated 38.4 million [33.9–43.8 million] HIV-positive individuals worldwide, with 25.6 million of them living in the WHO African Region [2].

In Ethiopia, the annual number of HIV infected people showed declining trends since 2002. Over the past two decades HIV prevalence rate decreased from 3.3% in 2000 to 0.9% in 2017, and AIDS-related deaths from 83,000 deaths in 2000 to 15,600 in 2017 [3].

More than 90% of pediatric AIDS cases are caused by vertical transmission from mother to child, which also accounts for the majority of heterosexual transmission in Ethiopia. According to a report from the Federal HIV/AIDS Prevention and Control Office, there are 65 new cases of the disease every day, 30 people die from it, and the MTCT has increased by 11% (60%–71%). HIV/AIDS transmission rates were 1.2% for females and 0.5% for males. Even when HCT services were provided, there was a discrepancy in their uptake throughout Ethiopia [4].

Many HIV-infected women rely on condoms, which don’t provide effective contraception [5, 6]. Male partner commitment and the negotiation of women for safer sex determines the consistent and correct use of condom [7]. Couples tend to use dual contraception if both partners are worried about unintended pregnancy and HIV or STI transmission [8] and whether the HIV-positive partner has disclosed his or her HIV status [5].

Women living with HIV appear to have a higher rate of unintended pregnancy (51–90%), compared to global estimates of other women (38%) [9].

Some women who are on Highly Active Antiretroviral Therapy (HAART) may use only highly effective contraceptive methods without condoms. In situations of poor adherence, this can increase the risk of transmission of drug resistant strains their partners [4, 10]. Therefore, the aim of this study was to assess dual contraception method utilization and associated factors among reproductive age women who were on antiretroviral therapy in Bishoftu Public health Facilities.

## Methods

A facility based cross sectional study was carried out from November 01 to December 30, 2020. The study was conducted in Bishoftu town health facilities. This was located at 47 Km to the South East of Addis Ababa. The town had 9 urban and 4 rural kebeles. In addition, the town had 1 general hospital, 5 public health centers, 26 private clinics, 2 non-governmental organization (NGO) clinics, 7 pharmacies, 5 drug stores and one drug vender. From the above health facilities, 1 general hospital and 1 health center were providing ART services. In the town, 6477 peoples were on Antiretroviral Therapy (ART). According to the report from Bushoftu town health bureau, in 2017; the number of HIV positive women in child bearing age group attending ART services in Bishoftu General hospital and Bishoftu health center were 1,171 and 417 respectively. The service was given by trained health workers in both health facilities providing the service.

The source population were all HIV positive women in reproductive age group (15–49 years) enrolled to ART units of Bishoftu general hospital and Bishoftu health center where as HIV positive women in reproductive aged group who were enrolled to ART during the time of data collection in both health facilities were study population.

All HIV positive women of reproductive age who attended the chronic HIV/AIDS care clinic at least once were included in the study, whereas women who were unable to communicate, those who were severely ill, and women with a confirmed pregnancy were excluded from this study.

Sample size was computed using single population proportion formula with the assumptions of 95% confidence level, 5% margin of error and the proportion of dual contraceptive utilization taken from study done in Fiche Hospital which is 32% [11]. Based on this, the sample size calculated was 334. Since the total source population was 1,588 (less than 10,000), finite population correction formula was used to reduce the sample size. After adding 10% non-response rate, the final sample size was determined to be 306.

Prior to data collection, lists of all HIV positive women in the child bearing age group attending the ART clinic were obtained from the ART registration book of both health facilities. A sampling frame was prepared using these lists and study participants (HIV positive women in child bearing age group) were selected by using computer generated simple random sampling techniques through an excel spread sheet. During the two-month study period; 306 HIV positive women were recruited from sampling frame. Respondents who did not come at their appointment date were revisited after they were contacted through phone calls and invited to come to the health facility.

Data were collected by face to face interview using structured, pre-tested Amharic version questionnaire. The questionnaire was initially prepared in English and translated to Amharic and back to English by language experts and researchers to keep the consistency of the questionnaire. Two well trained case managers who were working in the health facility collected the data and one health management information system professional had supervised the data collection process. Data collectors had cross checked the ART card numbers of all clients who came to the ART clinic with the sampled card numbers daily, the completed questionnaires were checked on-site for consistency and completeness daily by the supervisor and the principal investigators.

### Measurement

#### Dual contraceptive utilization

HIV positive women who used two methods of contraception simultaneously, a barrier method (male/female condom use in every sexual encounter in the last twelve months preceding the study) & other modern contraceptive methods (such as the IUCD, contraceptive implants, injections and pills) [12].

### Data analysis

After data collection, each questionnaire was checked for completeness and a code given before data entry. Then, data were entered in to Epi-data 3.1 and exported to statistical package for social science (SPSS) version 20.0 for analysis. Data were presented with mean with standard deviation for continuous variables and frequencies and percentages for categorical variables. Bivariable logistic regression analysis was conducted primarily to check if the variables had an association with the dependent variables. All variables associated with the dependent variables at a p value < 0.25 were entered in to a multivariable logistic regression model for controlling the possible effect of confounders and finally the variables which had significant associations with dual contraceptive utilization were identified on the basis of adjusted odds ratios with 95% CI & p-value of less than 0.05. Goodness of fit of the final model was checked using Hosmer & Lemeshow test considering good fit at P-value (<0.05).

### Ethics approval and consent to participate

Ethical clearance was obtained from the ethical review committee of the Adama Hospital Medical College. A permission letter was obtained from the Oromia Regional Health Bureau and the Bishoftu town administration health office. A letter of support was written to the town health office & health facilities to get permission before the start of the data collection. Finally, all the study participants were informed verbally about the purpose and benefit of the study along with their right to refuse and consent were obtained. Confidentiality of study participants were assured using identification number and removing names and other identifiers during the interview. Verbally informed consent were taken from all study participants.

## Results

### Socio-demographic characteristics of study participants

A total of 306 HIV positive women who were on chronic care and follow up participated in the present study with response rate of 100%. The mean age of participants was 33.84 years (±6.03SD years) and 164 (53.6%) study participants were found in the age group >35 years. The majority of the participants (85.6%) were urban residents. One hundred seventy-seven (57.8%) respondents were married and one hundred nineteen (38.9%) attended primary (1–8) education. Three-fourths of the participants were Orthodox Christian 228 (74.5%). One hundred seventeen (38.2%) were daily laborers and 79 (25.8%) were housewives (Table 1).

**Table 1:** Socio-demographic characteristics of HIV positive women in chronic care in Bishoftu Town Health institution, 2020 (n = 306).

Variable	Frequency	Percent
**Age (in years)** (mean = 33.84 years, SD±6.03SD)		
15–24	19	6.2
25–34	123	40.2
≥35	164	53.6
**Residenc**e		
Urban	262	85.6
Rural	44	14.4
**Marital Status**		
Married	177	57.8
Divorced/Separated	66	21.6
Widowed	31	10.1
Never married	32	10.5
**Educational Status**		
Primary (1–8)	119	38.9
Illiterate	84	27.5
Secondary (above9)	59	19.3
Read and write only	30	9.8
Technical/Vocational	3	1
Higher Education	11	3.6
**Religion**		
Orthodox	228	74.5
Muslim	8	2.6
Protestant	68	22.2
**Occupation**		
House Wife	79	25.8
Daily Laborer	117	38.2
Merchant	7	2.3
Private Employee	60	19.6
Government Employee	14	4.6
Unemployed	17	5.6
Farmer	8	2.6

### Awareness and utilization of dual method contraceptive

According to the present study, 258 (84.3%) ever heard about dual method contraceptives. Concerning source of information, 230 (75.2%) heard from health professionals. Regarding history of pregnancy, 56 (34.8%) of the pregnancies were unwanted since the start of chronic care follow up (Table 2).

**Table 2:** Awareness and utilization of dual method contraceptive among HIV positive women attending chronic care follow up in Bishoftu Town Health institution, 2020.

Variable	Frequency	Percent
Ever heard about contraceptive use (n = 306)		
Yes	302	98.7
No	4	1.3
Ever heard about Dual methods contraceptive use (n = 306)		
Yes	258	84.3
No	48	15.7
Heard Dual method from Health Professionals (n = 258)		
Yes	230	89.1
No	28	10.9
Heard Dual method from Radio (n = 258)		
Yes	8	3
No	250	97
Heard Dual method from Television (n = 258)		
Yes	20	7.8
No	238	92.2
Current Dual Method contraceptive users (n = 306)		
Yes	174	56.9
No	132	43.1
Ever got pregnancy since the start of chronic care (n = 306)		
Yes	145	47.6
No	161	52.4
Wanted pregnancy (n = 161)		
Yes	105	65.2
No	56	34.8

### Dual contraceptive utilization

**Fig 1:**
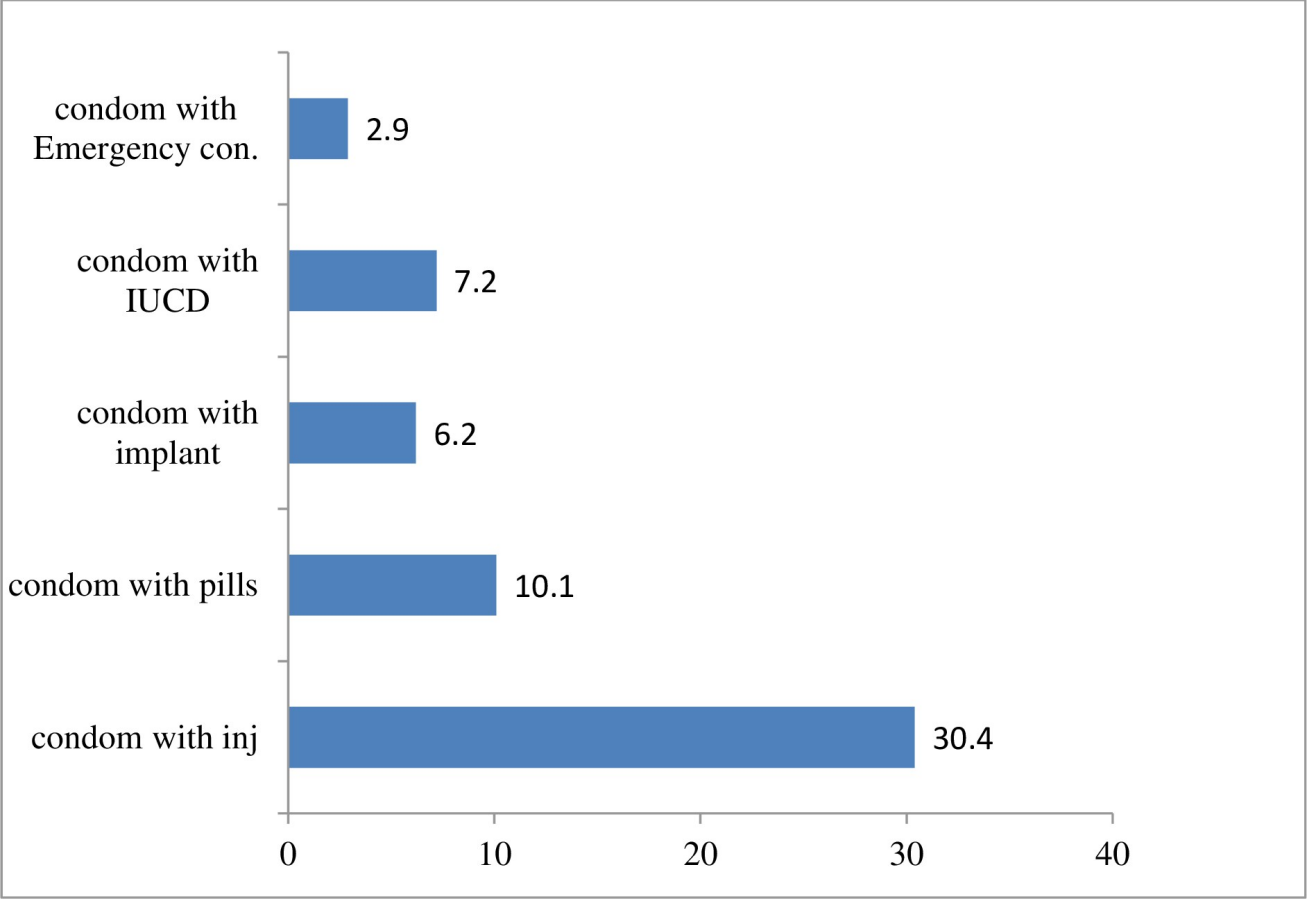
Types of dual contraceptive method used by HIV positive women attending chronic care follow up of Bishoftu Town Health institution, 2020.

The use of dual contraceptive utilization of among HIV positive women attending chronic care follow up in Bishoftu Town Health institution was 174 (56.8%). Of which, more than one-fourth (30.4%) had used condom with injectable (Fig 1).

### HIV status, disclosure and decision-making power

Considering partner’s HIV status, 85 (27.8%) of study participants didn’t know their husband’s/partner’s HIV status. From all study participants, 273 (89.2%) were informed about different contraceptive choices while 166 (54.2%) had desire to have children in the future. With regards to decision making, 168 (73.7%) participants had equal decision-making power with their partner on condom use (Table 3).

**Table 3:** Partner HIV status disclosure and discussion with partner and health care worker about contraceptive use among HIV positive women on chronic care follow up in Bishoftu Town Health institutions, 2020.

Variable	Frequency	Percent
Partner HIV status (n = 306)		
HIV positive	154	50.3
HIV negative	67	21.9
Unknown	85	27.8
Disclosure of HIV status (n = 306)		
Yes	204	66.7
No	102	33.3
Decision makers on condom use (n = 228)		
Husband/partner	26	11.4
Wife	34	14.9
Together	168	73.7
Ever told by service provider on method of contraceptive use (n = 306)		
Yes	273	89.2
No	33	10.8
Intention to have children in the future (n = 306)		
Yes	166	54.2
No	140	45.8
Intention on number of children (n = 166)		
1–2	60	36
3–4	24	15
> = 4	82	49

### Factors associated with dual method contraceptive use

In multivariable logistic regression analysis; factors significantly associated with using dual contraceptive method use were marital status, pregnancy since chronic care follow up and partner’s HIV status.

The odds of using dual contraceptive among married women were four times higher than widowed women (AOR = 4.33, 95% Cl; 1.67–11.27). Women who were not pregnant since the enrolment to chronic care had twice as higher odds of having used dual contraceptive to women who had been pregnant (AOR = 2.19, 95% C1; 2.90–3.70).

Women who have HIV positive partner had odds 2.67 times higher to use dual contraceptive than those with unknown partner’s HIV status (AOR = 2.67, 95% Cl; 1.34–5.32). The odds of using dual contraceptive among women with HIV negative partner were 2.38 times higher than those women whose partner’s HIV status was unknown (AOR = 2.38, 95% Cl; 1.09–5.20) (Table 4).

**Table 4:** Bivariable and multivariable logistic regression analysis of factors associated with dual method contraceptive utilization among HIV positive women on chronic care in Bishoftu Town Health institution, 2020.

Variable	Dual method contraceptive use		COR (95% Cl)	AOR (95% Cl)
	Yes No. (%)	No % No. (%)		
**Marital status**				
Married	128 (72.3)	49 (27.7)	7.51 (3.15–17.91)	4.33 (1.67,11.27)**
Single	11 (34.4)	21 (65.6)	1.5 (0.50–4.4)	1.87 (0.59–5.89)
Divorced	27 (40.9)	39 (59.1)	1.99 (0.77–5.10)	2.09 (0.77–5.67)
Widowed	8 (25.8)	23 (74.2)	1	1
**Ever give birth**				
Yes	158 (60.3)	104 (39.7)	2.6.6 (1.37–5.15)	1.73 (0.75,3.99)
No	16 (36.4)	28 (63.6)	1	1
**Ever got pregnancy since the start of chronic care follow up**				
Yes	132 (59.46%)	90 (40.54%)	1.92 (2.85–7.53)	2.19 (1.29,3.70)**
No	42 (43.30%)	55 (56.70%)	1	1
**Partner HIV status result**				
Positive	106 (68.83)	48 (31.17)	5.61 (3.13–10.05)	2.67 (1.34,5.32)**
Negative	44 (57.14)	23 (29.87)	4.86 (2.43–9.70)	2.38 (1.09,5.20)**
Not known	24 (28.23)	61 (71.75)	1	1

** Statistically significant at P-value <0.05

## Discussion

In the present study, the prevalence of dual method contraceptive use was 56.9%. This finding is higher than studies conducted in different parts of Ethiopia: (Gimbi 30%) [13] (Addis Ababa 18%) [14]. (Hosanna Hospital 28.3) [16]. The difference might be due to the difference in when the study was done. Similarly it is higher than in the study carried out in Kenya (38.5%) [15], south east Nigeria (27.2%) [16] and South Africa (33%) [17]. The difference might be due to the discussion that 89.1% of the women had with health professionals, regarding the benefits of dual contraception and other reproductive health needs. However, when compared to study conducted in north Ethiopia and Bahirdar prevalence of dual method in this study was lower, 59.9% and 64.2%, respectively [17, 18]. The difference might be due to difference in the role and commitment of care providers.

In this study, married women were more likely to use dual methods than widowed. These results were consistent with study conducted in north Ethiopia [17]. This might be due to the fact that married partners find it easier to discuss issues regarding contraception than unmarried partners. Partner disapproval and non-discussion with partner were among the reasons for non-use of dual methods Moreover, half of the dual method users belonged to a psychosocial support group where dual method use was highly advocated. This suggests that women may not make decisions to use contraceptives unilaterally, but this may occur in consultation with their social networks who can influence dual contraceptive use.

The proportion of women ever heard about dual method contraceptive was 84.9% and the major source of information were health care professionals. In this study, the awareness of dual method contraception was found to be higher than that of study conducted in north Ethiopia, which was 41.7% [17].

This might indicate the awareness of dual contraception method is increasing over the years possibly due to expansion of integrated family planning into the ART clinic as well as ongoing counselling on prevention of unintended pregnancy according to PMTCT guidelines in Ethiopia. Similarly, it was found to be higher when compared to South Africa study [19]. This might be due to geographical and study population variation.

Women whose partners were HIV negative were more likely to use dual contraception method when compared to women whose partners’ status were unknown. The finding is consistent with the findings from the study conducted in north Ethiopia, where women whose partners were HIV negative were more likely to use the dual contraceptive method as compared to women whose partners’ status were unknown. This might be due to the fact that those women who know their partners’ status are likely to be in committed relationships, therefore, better able to negotiate [17, 18].

According to this study, women who did not become pregnant since the start of the chronic care follow up were two times more likely to use dual contraception than women who got pregnant while on chronic care follow up. Among women who become pregnant, 34.3% reported the pregnancy was unintended indicating an unmet contraceptive need in this population. This could have been prevented by the dual contraception method. This present study revealed that the intention of HIV positive women to have a child in the future was 54.2%. This finding was found to be high when compared to study done in Fitche Hospital [11]. The reason might be due to the variation in study period and study area.

## Conclusions

The present study showed that the use of dual contraceptive methods was slightly lower compared to north Ethiopia and Bahirdar studies. Marital status, partner HIV status, pregnancy after chronic HIV care follow up were found to be significantly associated with dual contraceptive method utilization. Use of dual contraceptive methods utilization is essential in preventing unwanted pregnancy, STIs and transmission of resistant HIV strains among the study population. Besides, it needs governmental and non-governmental organization, professionals and researchers’ involvement to improve dual contraceptive utilization. Most frequently, family planning services are not integrated into HIV services; therefore, HIV-positive women typically must visit separate HIV clinics and reproductive health clinics to access both services. Integrating family planning services into HIV care may address some challenges faced by HIV-infected women in accessing their preferred contraceptive methods. The limitation of this study is that it has not covered a large area of Ethiopia but was conducted in a catchment area of a single town, hence the results may not be generalizable to the HIV infected women of child bearing age in Ethiopia.

## Supporting information

S1 File Data set. (XLSX)

S2 File English questionnaire. (DOCX)

S3 File Amharic questionnaire. (DOCX)

## List of abbreviations

AIDS: Acquired Immunodeficiency Syndrome
AOR: Adjusted Odds Ratio
ART: Antiretroviral Treatment
HIV: Human Immuno-deficiency Virus
MTCT: Mother to child Transmission
HAART: Highly Active Antiretroviral Therapy
NGO: Non-governmental Organization
STIs: Sexually Transmitted Infections

## References

[1] Wilson TE, Koenig LJ, Walter E, Fernandez I, Ethier K. Perinatal Guidelines Evaluation P. Dual contraceptive method use for pregnancy and disease prevention among HIV-infected and HIV-uninfected women: the importance of an event-level focus for promoting safer sexual behaviors. Sex Transm Dis. 2003; 30 (11):809–12. 14603086 10.1097/01.OLQ.0000086617.41012.14

[2] World Health Organization. Fact Sheets on HIV. 9^th^ November, 2022. Available at https://www.who.int/news-room/fact-sheets/detail/hiv-aids.

[3] HAPCO. HIV prevention in Ethiopia National Road map; 2018. Available at *https://ethiopia.unfpa.org/en/…/hiv-prevention-ethiopia-national-road-map*.

[4] Federal Democratic Republic of Ethiopia HIV Prevention and Control Office, Country Progress Report on the HIV Response, Federal Democratic Republic of Ethiopia HIV Prevention and Control Office, Addis Ababa, Ethiopia, 2011–2014. Available at https://www.google.com/search?q=Federal+Democratic+Republic+of+Ethiopia+HIV+Prevention+and+Control+Office%2C+Country+Progress+Report+on+the+HIV+Response%2C+Federal+Democratic+Republic+of+Ethiopia+HIV+Prevention+and+Control+Office%2C+Addis+Ababa%2C+Ethiopia%2C+20112014&oq=Federal+Democratic+Republic+of+Ethiopia+HIV+Prevention+and+Control+Office%2C+Country+Progress+Report+on+the+HIV+Response%2C+Federal+Democratic+Republic+of+Ethiopia+HIV+Prevention+and+Control+Office%2C+Addis+Ababa%2C+Ethiopia%2C+20112014&aqs=chrome..69i57.408j0j7&sourceid=chrome&ie=UTF-8.

[5] Haddad L, Feldacker C, Jamieson D, Tweya H, Cwiak C, Chaweza T et al. Pregnancy prevention and condom use practices among HIV-infected women on antiretroviral therapy seeking family planning in Lilongwe, Malawi. PLoS One. 2015; 10(3). doi: 10.1371/journal.pone.0121039 25811849 PMC4374940

[6] Joshi Beena, Velhal Gajanan, Chauhan Sanjay, Kulkarni Ragini, Begum Shahina. Contraceptive use and unintended pregnancies among HIV-Infected women in Mumbai. Indian J Community Med. 2015; 40(3):168–173. doi: 10.4103/0970-0218.158855 26170540 PMC4478657

[7] Nakaie Naomi, Tuon Sovanna, Nozaki Ikuma, Yamaguchi Fuzuki, Sasaki Yuri, Kakimoto Kazuhiro. Family planning practice and predictors of risk of inconsistent condom use among HIV-positive women on anti-retroviral therapy in Cambodia. BMC Public Health. 2014;14(1):170. doi: 10.1186/1471-2458-14-170 24528885 PMC3936956

[8] Melaku Yohannes, Zeleke Ejigu Gebeye. Contraceptive utilization and associated factors among HIV positive women on chronic follow up care in Tigray Region, Northern Ethiopia: a cross sectional study. PLoS One. 2014; 9(4). doi: 10.1371/journal.pone.0094682 24743241 PMC3990566

[9] The Federal Democratic Republic of Ethiopia. Ministry of Health. Country progress report on the HIV response. 2014.

[10] Fedral Minstry of Health. National Guidelines For Comprehensive Hiv Prevention, Care And Treatment. 2017.

[11] Demissie DB, Girma T, Abdissa G. Dual contraceptive utilization and associated factors among people living with HIV attending ART clinic in Fitche Hospital Ethiopia. J Health Med Nurs. 2015;20:2422–8419.

[12] Jifar MS, Handiso TB, Mare TD, Ibrahim SA. Dual Contraceptive Utilization and Associated Factors among Human Immunodeficiency Virus (HIV) Positive Women Attending Anti Retro Viral Therapy (ART) Clinic in Hossana Hospital, Southern Ethiopia. 2017; 3(2): 1023.

[13] Polisi A, Gebrehanna E, Tesfaye G and Asefa F. Modern contraceptive utilization among female ART attendees in health facilities of Gimbie town, West Ethiopia. Reproductive Health. 2014; 11:30. doi: 10.1186/1742-4755-11-30 24731751 PMC3989849

[14] Asfaw HM & Gashe FE. Contraceptive use and method preference among HIV positive women in Addis Ababa, Ethiopia: a cross sectional survey. *BMC Public Health*. 2014: 14 **(**566). 10.1186/1471-2458-14-566 PMC4063250 24902478

[15] Mulongo AM, Lihana RW, Githuku J, Gura Z and Karanja Simon. Factors associated with uptake of dual contraception among HIV-infected women in Bungoma County, Kenya: a cross-sectional study. Pan Afr Med. 2017;28(1):2. doi: 10.11604/pamj.supp.2017.28.1.9289 30167030 PMC6113694

[16] Lawani LO, Onyebuchi AK. & Iyoke CA. Dual method use for protection of pregnancy and disease prevention among HIV-infected women in South East Nigeria. BMC Women’s Health. 2014;14(39):1–8. doi: 10.1186/1472-6874-14-39 24602410 PMC3973850

[17] Gebrehiwot SW, Azeze GA, Robles CC, Adinew YM. Utilization of dual contraception method among reproductive age women on antiretroviral therapy in selected public hospitals of Northern Ethiopia. Reprod Health. 2017; 14 (25). doi: 10.1186/s12978-017-0390-6 28982364 PMC5629799

[18] Kebede H, Nahusenay H, Birhane Y. and Tesfaye D. Assessment of Contraceptive Use and Associated Factors among HIV Positive Women in Bahir-Dar Town, Northwest Ethiopia. Open Access Library Journal. 2015; 2(10): 1–19.

[19] Osuafor GN, Maputle SM. Dual Protection and Contraceptive Method Use among Women in Heterosexual Relationships in Mahikeng, South Africa. Afr J Reprod Health. 2017; 21(1):64–72. doi: 10.29063/ajrh2017/v21i1.5 29595026

