# Supplementary material for: Dual contraception method utilization and associated factors among women on anti-retroviral therapy in public facilities of Bishoftu town, Oromia, Ethiopia
Source: PLoS One. 2023 Jan 17;18(1):e0280447. doi: 10.1371/journal.pone.0280447 (PMC9844831; doi:10.1371/journal.pone.0280447)
Supplement: S2 File — (DOCX) [file pone.0280447.s002.docx]

# ANNEXES II: ENGLISH QUESTIONNAIRE

Dual methods Contraceptive Utilization and Associated factors among HIV positive women on chronic care follow up in public Health Institutions of Bishoftu Town, Oromia, Ethiopia, 2020.

| Study unit code _____________ | Date_________________ |
| --- | --- |
| Name of Health institution | Bishoftu Hospital & Bishoftu health center |
| Name of data collector |  |
| Signature |  |

**Section 1**: Socio demographicinformation

I’d like to start by asking you some general questions about your daily life. Are you ready to begin?

| NO | Question | Coding categories |  |
| --- | --- | --- | --- |
| 101 | How old are you? | _________ years |  |
| 102 | What is your residence? | 1. Urban 2. Rural |  |
| 103 | What is your current marital status? | 1. Married 2. Single 3. Divorced/separated  4.Widowed 5.Other |  |
| 104 | What is the highest level of education you attended? | Illiterate  Read and write only  Primary (1-8)  Secondary (above 9)  Technical/vocation  Higher education |  |
| 105 | What is your religion? | 1. Orthodox 2. Muslim  3. Protestant 4. Catholic  5. other __________________ |  |
| 106 | To which ethnic group do you belong? | 1. Oromo 2. Amhara  3. Tigre 4.Other __________ |  |
| 107 | What is your occupation at this time? | House wife  Daily laborer  Student  Unemployed  Private employee  Government employee  Merchant  Others (specify)--------------- |  |
| 108 | What is your average monthly household income (total wages/income earned by all living with you)? | _____________ birr |  |
| 109 | Have you ever given birth? | 1. Yes 2. No | If No, 201 |
| 110 | How many Biological child/children do you have? | ----------------- |  |

**Section** 2: Awareness and current use of dual method contraceptive by HIV positive women

Now I am going to ask you questions about knowledge and your current use of family planning methods

| No | | | Question | | Coding categories | |  |  | |
| --- | --- | --- | --- | --- | --- | --- | --- | --- | --- |
| 201 | | | Have you ever heard about contraceptive use? | | 1. YES 2. NO | |  |  |  |
| 2 202 | | | Have you ever got pregnancy since you s started Chronic care follows up? | | 1. Yes 2. No | | If yes, 203,204 |  |  |
| 203 | | | Was the pregnancy wanted? | | 1. Yes 2. No | |  |  |  |
| 204 | | | What was the outcome of the pregnancy? | | 1. abortion 2. still birth 3. live birth 4. other | |  |  |  |
| 205 | | | Have you ever heard of sexually Transmitted Infections (STIs), other than HIV/AIDS? | | 1. Yes 2. no | | If yes, 206  If No,208 |  |  |
| 206 | Which methods have you ever heard to protect yourself from Sexually Transmitted Infections, including HIV? | | 1. Pill 2. IUD 3. Male condom 4. Female condom 5. Implants 6. Injectables 7. Emergency hormonal contraception 8. Female sterilization / tubal ligation 9. Periodic abstinence 10. Other (specify) ________ 11. No Response | |  | | |  |  |
| 207 | Which method have you been using to prevent STIs? | | 1. Pill 2. IUD 3. Male condom 4. Female condom 5. Implants 6. Injectables 7. Emergency hormonal contraception 8. Female sterilization / tubal ligation 9. Periodic abstinence 10. Other (specify) ________ 11. No Response | |  | | |  |  |
| 208 | Have you ever heard of using condom with other contraceptive methods (like condom with injection) at the same time? | | 1. YES 2. NO | | If yes,209,  210 | | |  |  |
| 209 | From where have you heard about using condom with other contraceptives? | | Health professionals  Radio  Television  Other (specify)----------- | |  | | |  |  |
| 210 | Have you been using condom with another contraceptive method (like condom with injection) at the same time in the last 12 months? | | 1. YES 2. NO | | If yes, 211,212  If No, 215 | | |  |  |
| 211 | Why did you do this?  *(CIRCLE ALL MENTIONED)* | | 1. to prevent STIs 2. to prevent HIV transmission 3. to delay or prevent pregnancy 4. to prevent both pregnancy and infections 5. other(specify)_______ | |  | | |  |  |
| 212 | Which method have you been using in the last 12 month? | | 1. condom with Pill 2. condom with IUD 3. Male condom only 4. condom with Implants 5. condom with Injectables 6. condom with Emergency hormonal contraception 7. condom with Female sterilization / tubal ligation 8. condom with Periodic abstinence 9. Other (specify) ________ | | If yes, 215 | | |  |  |
| 213 | Since what month and year have you been using (CURRENT MOTHOD) without stopping?  PROBE: For how long have you been using (CURRENT METHOD) now without stopping? | | Month -------------(MM)  Year --------------(YYYY) | |  | | |  |  |
| 214 | Where did you obtain (CURRENT METHOD) the last time? | | Govt. Hospital  Govt. health center  Govt. health post  Private Hospital  Private clinic  Private drug vendor/store/ shop  NGO health facility | |  | | |  |  |
| 215 | What are the reasons you are not using a methods? | | 1. Hysterectomy 2. Wants more children now 3. Fears side effects 4. Inconvenient to use 5. infrequent sex 6. Husband opposed 7. Religious prohibition 8. Other (specify) ________ | |  | | |  |  |
| 216 | Have you used condom in your last 12 month during sex? | | 1. Yes 2. no | | If yes,217  If no,218 | | |  |  |
| 217 | How frequently have you used condoms during sexual intercourse in the last six month? | | Always (100% of the time)  Most times (> half of the time)  Some times (about half of the time)  Rarely (< half of the time)  Never (No condom use)  other­­ (specify)_________ | |  | | |  |  |
| 218 | For you / in your relationship, what are the main problems with using a condom during sex? | | Difficult to get condoms  Condoms not comfortable for me/partner  Condoms not necessary in current relationship  Cannot tell partner to use condoms   1. Other (Specify)_____ | |  | | |  |  |
| 219 | Do you think that using a condom alone would be effective in preventing both pregnancy and STIs? | | Yes  No | |  | | |  |  |
| 220 | Since what month/year you started chronic care follow up? | | ----------------(months) | |  | | |  |  |
| 221 | What is your ART status? | | 1. On ART  2. On Pre-ART | |  | | |  |  |

**Section 3**: Partner HIV status, Disclosure and discussion with partner and/or health care worker about contraceptive use

Now I am going to ask you about your partner HIV status and discussion with him and Health professional about contraceptive use

| NO | Question | Coding categories |  |
| --- | --- | --- | --- |
| 301 | What is your partner HIV status? | HIV positive  HIV negative  not known |  |
| 302 | Have you disclosed your HIV status to your partner? | yes  no | If yes,304 |
| 303 | Have you ever talked about using a condom with another contraceptive method at the same time with your partner? | Yes  No |  |
| 304 | Who in the couple should make decisions about whether or not to use a condom? | husband/ partner  wife  together |  |
| 305 | Were you ever told by a health or family planning worker about other methods of contraceptive that you could use? | yes  no | If yes,306,307 |
| 306 | Which method would you prefer to use?  (*CIRCLE ALL MENTIONED )* | 1. Pill 2. IUD 3. Male condom 4. Implants 5. Injectables 6. Emergency hormonal contraception 7. Female sterilization / tubal ligation 8. Periodic abstinence 9. Dual method   Other |  |
| 307 | Why you choose dual method? | Health professionals advice  Perceived less side effects  Observed friends experience  Easy to use  Agreement with partner  Effectiveness  other (specify) _______ |  |
| Q309 | Are you using the method because you want to have another child later or because you want no more children at all? | Wants another child later 1 Wants no more children 2 No Response 9 | |
|  |  |  |  |

**Section 4**: Intention to have child in the future

| No | Questions | Coding categories |  |
| --- | --- | --- | --- |
| 401 | Do you want to have a baby in the future? | 1. Yes 2. No 3. No response | If yes,402,  403 |
| 402 | When do you want to have your next baby? | 1. Within the next 12 months 2. Within 1-2 years 3. After 2 years 4. After I marry 5. When God wants 6. Other(specify) ___ 7. No Response |  |
| 403 | How many children would you like to have in the future? | 1. 1 2. 2 3. 3 4. >=4 5. no response |  |
