# Supplementary material for: Dual contraception method utilization and associated factors among women on anti-retroviral therapy in public facilities of Bishoftu town, Oromia, Ethiopia
Source: PLoS One. 2023 Jan 17;18(1):e0280447. doi: 10.1371/journal.pone.0280447 (PMC9844831; doi:10.1371/journal.pone.0280447)
Supplement: S3 File — (DOCX) [file pone.0280447.s003.docx]

# ANNEX III: AMHARIC QUESTIONNAIRE

የአማርኛቃለመጠይቅ

**ምዕራፍ 1**፡ማህበራዊመረጃዎች: መሰረታዊ የማሀበራዊ መረጃዎችን በመጠየቅ እጀምራለዉ፡፡ ዝግጁ ኖት?

| የጥያቄኮድቁጥር | ጥየቄ | ምርጫ |  |
| --- | --- | --- | --- |
| 101 | እድሜዎት ስንት ነዉ? | ---------ዓመት |  |
| 102 | የመኖሪያ ቦታ የት ነዉ? | ገጠር 2. ከተማ |  |
| 103 | የጋብቻ ሁኔታዎ ምን ይመስላል? | ያገባች  ያላገባች  የተፋቱ/የተለያዩ  ባልየሞተባት  ለላ (ይግለፁ)-------------- |  |
| 104 | የትምርት ደረጃዎ ስንትነዉ? | ምንምያልተማረች  መጻፍናማንበብብቻ  የመጀመሪያደረጃ (1-8 ክፍል)  ሁለተኛደረጃ (9ኛናከዛበላይ)  ቴክንክናሙያ  ከፋተኛትምርት |  |
| 105 | የምንሀይማኖትተከታይኖት? | ኦርቶዶክስ  እስልምና  ፕሮቴስታንት  ካቶሊክ  ልላ (ይግጹ)------------ |  |
| 106 | የርስዎብሄርየትኛዉነዉ? | ኦሮሞ  አማራ  ትግሬ  ልላ (ይግለጹ)------------ |  |
| 107 | በአሁኑሰዓትስራዎትምንድነዉ? | የቤትእመቤት  የቀንሰራተኛ  ተማሪ  ስራአጥ (ስራአልያዙም)  የግልተቋምሰራተኛ  የመንግስትተቋምሰራተኛ  ሌላ (ይግከጹ)------------ |  |
| 108 | በአማካይየወርገቢዎ (ከርስዎጋርየሚኖሩትንጨምሮ) ምንያህልነዉ? | ­­­­­­­­­­­­­­­­­­  --------------ብር |  |
| 109 | ልጅአለዎት? | አዎ  አይ | አዎከሆነ፣110 |
| 110 | እርስዎየወለዱትስንትልጅአለዎት? | ------------------ |  |

**ምዕራፍ 2**፡ከኤችአይቪቫይረስጋርየሚኖሩትሴቶችስለጥምርየእርግዝናመከላከያዘዴ (ኮንዶምናሌላዘመናዊየእርግዝናመከላከያዘዴበጋራ )ያላቸዉግንዛቤናየመጠቀምሁኔታያለበትደረጃአሁንስለቤተሰብእቅድእርግዝናመከላከያዘዴ) ያለዎትግንዘቤ (እዉቀት) እናበአሁኑሰዓትስለምጠቀሙትዘዴእጠይቅዎታለዉ፡

| የጥያቄኮድቁጥር | ጥየቄ | ምርጫ |  |
| --- | --- | --- | --- |
| 201 | ስለእርግዝናመከላከያሰምተዉያቃሉ? | አዎ  አይ | መልሱአዎከሆነ፣ወደጥያቄ 202 እና 203 |
| 202 | የፀረኤችአይቪ/ኤድስህክምናክትትልከጀመሩቦሃላአርግዘዉያቃሉ? | አዎ 2. አይ |  |
| 203 | እርግዝናዉበእቅድነበር (የተፈለገነበር)? | አዎ 2. አይ |  |
| 204 | የእርግዝናዉዉጤትምንነበር? | ዉርጃ  ሞቶየተወለደ  በሀይወትየተወለደ  ሌላ (ይግለፁ)------------ |  |
| 205 | ስለአባለዘርበሽታ (ኤችአይቪንሳይጨምር) ሰምተዉያቃሉ? | አዎ  አይ |  |
| 206 | ከሚከተሉትዉሰጥየትኛዉየአበላዘርበሽታንለመከላከልይረዳል?  (የተጠቀሰዉንሁሉያክብቡ) | የእርግዝናመከላከያእንክብል (ፒልስ)  ሉፕ( IUD)  የወንድኮንዶም  የሴትኮንዶም  በመርፌየሚሰጥ(ዲፖፐሮቬራ)  ድንገተኛየእርግዝናመከላከያ  ማሀፀንማስቋጠር / tubal ligation  የወንድዘርፍሬንማምከን/ / vasectomy  መታቀብ  ቆዳስርየሚቀበር( implants)  ሌላ (ይግለጹ)------------------  መልስአልመለሱም |  |
| 207 | አባላዘርበሽታንለመከላከልእርስዎየትኛዉንዘዴእየተጠቀሙይገኛሉ? | የእርግዝናመከላከያእንክብል (ፒልስ)  ሉፕ( IUD)  የወንድኮንዶም  የሴትኮንዶም  በመርፌየሚሰጥ(ዲፖፐሮቬራ)  ድንገተኛየእርግዝናመከላከያ  ማሀፀንማስቋጠር / tubal ligation  የወንድዘርፍሬንማምከን/ / vasectomy  መታቀብ  ቆዳስርየሚቀበር( implants)  ሌላ (ይግለጹ)------------------  መልስአልመለሱም |  |
| 208 | ስለጥምርዘዴ፤ኮንዶምከሌላዘመናዊየእርግዝናመከላከያጋር (ምሳሌ፡ኮንዶምናመርፌበጋራ) ሰምተዉያቃሉ? | አዎ  አይ |  |
| 209 | ከየትእንደሰሙይንገሩን? | ከጤናባለሙያ  ከቴሌቪዥን  ከሬድዮ  ከሌላ (ይግለጹ)------------- |  |
| 210 | በአሁኑወቅትጥምርዘዴ፤ኮንዶምከሌላዘመናዊየእርግዝናመከላከያጋር (ምሳሌ፡ኮንዶምናመርፌ) ተጠቅመዉያቃሉ? | አዎ  አይ |  |
| 211 | ጥምርዘዴን፤ኮንዶምከሌላዘመናዊየእርግዝናመከላከያበጋርመጠቀምለምንይጠቅማልብለዉያስባሉ? | የአባለዘርበሽታንለመከላከል  ኤችአይቪንለመከላከል  እርግዝናንለመከላከል  እርግዝና፤አባላዘርበሽታናኤችአይቪንአንድላይለመከላከል  ሌላ (ይግለጹ)--------- |  |
| 212 | ከሚከታሉትዉስጥየትኛዉንጥምርዘዴንየተጠቀሙት/እየተተጠቀሙይገኛሉ? | ኮንዶምናየእርግዝናመከላከያእንክብል (ፒልስ)  ኮንዶምናሉፕ( IUD)  ኮንዶምናበመርፌየሚሰጥ(ዲፖፐሮቬራ)  ኮንዶምናድንገተኛየእርግዝናመከላከያ  ኮንዶምናማሀፀንማስቋጠር / tubal ligation  ኮንዶምናቆዳስርየሚቀበር( implants)  ሌላ (ይግለጹ)------------------  መልስአልመለሱም |  |
| 213 | አሁንእየተጠቀሙበትያሉትንጥምርዘዴሳያቋርጡለምንያህልግዜተጠቅᎂል? | -----------ወር  ------------ዓመት |  |
| 214 | አሁንእየተጠቀሙበትያሉትንጥምርዘዴየመጨረሻዉንከየትነዉየወሰዱት? | ከመንግስትሆሰፕታል  ከመንግስትጤናጣቢያ  ከመንግስትጤናኬላ  ከግልሆስፕታል  ከግልፋርማሲ  መንግስታዊካልሆነጤናድርጂት  ሌላ (ይግለጹ)----------- |  |
| 215 | ጥምርየእርግዝናመከላከያዘዴንእንዳይጠቀሙያረገዎትምክንያትምንድነዉ? | መሀፀንበማስቋጠርምክንያት  ልጅመዉለድእፈልጋለዉ  ከፀረኤችአይቪመድሀኒትጋርስለማይስማማ  ተጓዳኝችግሮችንበመፍራት  ስለማይመች  የግብረስጋግንኙነትስለማላዘዎትር  የድህረወሊድግዜላይስለሆንኩ (እስከ 6 ሳምንት)  ጡትእያጠባዉስለሆነ  ባለቤቴ (ባል) አይፈቅድም  ሀይማኖቴአይፈቅድም  ሌላ (ይግለፁ)--------------  መልስአልመለሱም |  |
| 216 | ባለፉት 12 ወራትበግብረስጋግንኙነትወቅትኮንዶምንተጠቅመዉያቃሉ? | አዎ  አይ |  |
| 217 | በለፉት 6 ወራትመቼመቼይጠቀሙነበር? | ሁልግዜ (100%)  አብዛኛዉንግዜ (ከግማሽግዜበላይ)  አልፎአልፎ (ከግማሽግዜበታች)  ምንምአልተጠቀምኩም  ሌላ (ይግለፁ)--------- |  |
| 218 | በርስዎናበባለቤትዎመካከልበግብረስጋግኑኝንትወቅትኮንዶምእንዳይጠቀሙምክንያት/ችግሮችምንድናቸዉ? | ኮንዶምማግኘትያስቸግራል  ምቾትአይሰጥም  በትዳርዉስጥኮንዶምአያስፈልግም  ለባለቤቴመንገርአልችልም  ሌላ (ይግለጹ)------------- |  |
| 219 | እርግዝናንናናየአባለዘርበሽታንለመከላከልኮንዶምመጠቀምብቻበቂይመስሎታል? | አዎ  አይ |  |
| 220 | የፀረኤች.አይ. ቪህክምናመከታተልከጀመሩምንያህልግዜይሆናለል? | ----------ወር  -----------ዓመት |  |
| 221 | የፀረኤች.አይ. ቪመድሀኒት (ART)  ጀምሯል? | አዎ  አይ |  |

**ምዕራፍ 3**፡የትዳርጓደኛ/ባል/የፍቅርጓደኛኤችአይቪዉጤት፤እናስለቤትሰብእቅድከትዳርጓደኛ/ባል/ጓደኛናከጤናባለሙያያለዉዉይይትያለበትደረጃ፡

| የጥያቄኮድቁጥር | ጥየቄ | ምርጫ |  | | |
| --- | --- | --- | --- | --- | --- |
| 301 | ባለቤትዎ/የፍቅርጓደኛዎየኤችአይቪዉጤትሚንይመስላል? | ኤችአይቪፖዘቲቭ  ኤችአይቪነገቲቭ  አይታወቅም |  | | |
| 302 | የኤችኤይቪቫይረስበደምዎዉስጥመኖሩንለባለቤትዎ/ፍቅርጓደኛዎነግረዋል? | አዎ  አይ | መልሱአዎከሆነ፤ 304 | | |
| 303 | ስለጥምርእርግዝናናእነፌክሽንመከላከያዘዴመጠቀምንበተመለከተከባለቤትዎ/የፍቅርጓደኛጋተወያይቶያዉቃሉ? | አዎ  አይ |  | | |
| 304 | ከርስዎናባለቤትዎ/የፍቅርጓደኛኮንደምንለመጠቀም/ላለመጠቀምማነዉየሚወስነዉ? | ባል/የፍቅርጓደኛ  ሚስት  ሁለቱምበጋራ |  | | |
| 305 | የጤናባለሙያዎችየቤተሰብእቅድንእንዲጠቀሙመክረዉየዉቃሉ? | አዎ  አይ | | መልሱአዎከሆነ፤ 306 |  |
| 306 | እርስዎየተኛዉንየቤተሰብእቅድ/ወሊድመከላከያመጠቀምይፈልጋሉ? | ኮንደምከሌላዘመናዊየእርግዝናመከላከያጋር  ሉፕ( IUD)  የወንድኮንዶም  የሴትኮንዶም  በመርፌየሚሰጥ(ዲፖፐሮቬራ)  ድንገተኛየእርግዝናመከላከያ  ማሀፀንማስቋጠርመታቀብ  ቆዳስርየሚቀበር  መልስአልመለሱም |  | | |
| 307 | ይሄንንዘዴለምንመረጡ? | በጤናባለሙያምክር/እገዛነዉ  አነስተኛተጓዳኝችግርይኖረዋልብየስላሰብኩ  ከጓደኞቼበማየት  አጠቃቀሙስለሚመች  ከባለቤቴጋርስለተስማማንበት  ዉጤታማዘዴስለሆነ  ሌላ (ይግለጹ) |  | | |

**ምዕራፍ 4**፡ወደፊትልጅንስለመዉለድበተመለከተ

| የጥያቄኮድቁጥር | ጥየቄ | ምርጫ |  |
| --- | --- | --- | --- |
| 401 | ወደፊትልጅመዉለድይፈልጋሉ? | አዎ  አይ  መልስአልመለሱም | መልሱአዎከሆነ፤ 402፣ 403 |
| 402 | መቼመዉለድይፈልጋሉ? | በሚቀጥሉት 12 ወራት  በሚቀጥሉት 1-2 አመታት  ከ2 ዓመትቦሃላ  ካገባሁቦሃላ  እግዝአብህርስፈቅድ  ሌላ(ይግለፁ)--------------  መልስአልተመለሰም |  |
| 403 | ስንትልጅእንድኖሮትይፈልጋሉ? | 1. 1 2. 2 3. 3 4. 4 5. >4 6. መልስ አልተመለሰም |  |

ቃለመጠይቁተጠናቋል፡፡ግዜዎን ስለሰጡንከልብእናመሰግናለን!
